# Addressing the environmental sustainability of eye health-care delivery: a scoping review

**DOI:** 10.1016/S2542-5196(22)00074-2

**Published:** 2022-06-01

**Authors:** John C Buchan, Cassandra L Thiel, Annalien Steyn, John Somner, Rengaraj Venkatesh, Matthew J Burton, Jacqueline Ramke

**Affiliations:** International Centre for Eye Health, London School of Hygiene & Tropical Medicine, London, UK; NYU Grossman School of Medicine, Department of Population Health, NYU Langone Health, New York, NY, USA; Department of Opthalmology, Groote Schuur Hospital, University of Cape Town, Cape Town, South Africa; Department of Opthalmology, Cambridge University Hospitals NHS Foundation Trust, Cambridge, UK; Aravind Eye Hospital, Pondicherry, India; International Centre for Eye Health, London School of Hygiene & Tropical Medicine, London, UK, National Institute for Health Research Biomedical Research Centre for Ophthalmology at Moorfields Eye Hospital NHS Foundation Trust and UCL Institute of Ophthalmology, London, UK; International Centre for Eye Health, London School of Hygiene & Tropical Medicine, London, UK, School of Optometry and Vision Science, University of Auckland, Auckland, New Zealand

## Abstract

The demand for eye care—the most common medical speciality in some countries—is increasing globally due to both demographic change and the development of eye health-care services in low-income and middle-income countries. This expansion of service provision needs to be environmentally sustainable. We conducted a scoping review to establish the nature and extent of the literature describing the environmental costs of delivering eye-care services, identify interventions to diminish the environmental impact of eye care, and identify key sustainability themes that are not yet being addressed. We identified 16 peer-reviewed articles for analysis, all published since 2009. Despite a paucity of research evidence, there is a need for the measurement of environmental impacts associated with eye care to be standardised along with the methodological tools to assess these impacts. The vastly different environmental costs of delivering clinical services with similar clinical outcomes in different regulatory settings is striking; in one example, a phacoemulsification cataract extraction in a UK hospital produced more than 20 times the greenhouse gas emission of the same procedure in an Indian hospital. The environmental costs must be systematically included when evaluating the risks and benefits of new interventions or policies aimed at promoting safety in high-income countries.

## Introduction

Climate change is regarded by many as the greatest long-term threat to global health in the 21st century.^1^ Although modest progress has been made by many sectors, overall, global greenhouse gas emissions continue to rise.^2,3^

Health-care services are substantial contributors to national greenhouse gas emission totals, representing 10% in the USA,^4^ 7% in Australia,^3^ 5% in Canada^5^ and Japan,^6^ and 4% in the UK.^7^ A 2019 report estimated that health-care emissions contributed 4·4–5·0% of all global greenhouse gas emissions (excluding African countries).^8,9^ If global health care were a country, it would be the fifth largest contributor to carbon emissions in the world.^8^ Eye care, as a high-volume service, probably forms a substantial part of these emissions. For example, in the UK, ophthalmology is the highest volume speciality, accounting for 8·1% of hospital outpatient visits nationally in 2018–19.^10^

The adverse effects of eye health-care delivery on the environment will probably increase. Ongoing innovation is broadening the range of interventions available, and demand is increasing as the world population grows and ages. Even in high-income countries (HICs) where one cataract operation is typically done per 100 population each year,^11^ demographic shifts are expected to increase case numbers of major ophthalmic conditions over the next 20 years, with projected increases for the UK of 52% for cataracts, 49% for glaucoma, and 64% for neo-vascular age-related macular degeneration.^12^ Far greater increases are expected in low-income and middle-income countries (LMICs), which have a greater capacity for demographic shift and development of health services.^13^ Consequently, the environmental impact of eye health services will continue to increase unless substantial changes are made to current practice.

UN Sustainable Development Goals (SDGs) were developed in 2015 as the overarching international strategic agenda. Several SDGs intersect with the way we deliver health care, including: SDG3 on Good Health and Wellbeing, SDG12 on Responsible Consumption and Production, and SDG13 on Climate Action.^14,15^ WHO published guidance on increasing the environmental sustainability of health-care facilities in October, 2020, but the guidance, although welcome, was not able to cite any peer-reviewed evidence from trials of proposed interventions to promote environmental sustainability.^16^ Similarly, a 2021 scoping review reporting interventions to improve the quality of cataract surgery, proposed the addition of planetary health to the seven dimensions of quality accepted by WHO, but was unable to identify any study targeting this aspect of quality in cataract service delivery.^17,18^ As recently as 2010 the opinion can be found in ophthalmic peer-reviewed literature debating this issue that “patient care should […] govern future healthcare policy, rather than the fashionable ‘carbon footprint’ lobby.”^19^

As a starting point, it is important to comprehensively understand what is currently known about this issue. Therefore, as part of *the Lancet Global Health Commission on Global Eye Health*,^20^ we undertook a Scoping Review to establish the nature and extent of the literature describing the environmental costs of delivering eye-care services, interventions to diminish these impacts, and to identify key sustainability themes that are not currently being addressed.

## Methods

### Search Strategy and Selection Criteria

Environmental sustainability in eye-care service delivery is a new field of study. We anticipated that the literature would be heterogeneous and sparse. Therefore, we selected a Scoping Review method to assess its nature and extent and we report findings according to the relevant sections of the Preferred Reporting Items for Systematic Reviews and Meta-analyses (PRISMA) Extension for Scoping Reviews guidelines.^21^ Our protocol was registered on the Open Science Framework repository. A Cochrane eyes and vision information specialist conducted searches in MEDLINE, Embase, and Global Health databases on Feb 1, 2022. The search broadly combines terms about eye health with terms about environmental impact and sustainability. The full search strategies are included in the appendix. No time, language, or geographic limits were applied. Reference lists of all included articles were examined to identify further potentially relevant studies.

We included observational studies of the environmental impacts of eye health care or manufacturing-related products (quantified as carbon dioxide equivalents [CO_2_e] or other measures of greenhouse gas emission, air or water toxins or other pollutants, or indirectly by energy consumption), interventional studies investigating strategies to diminish the environmental impacts of eye health-care provision, and literature reviews or modelling exercises that explore either the environmental impacts of eye health care or interventions to mitigate impacts which report eye care specifically.

We excluded studies of waste (eg, wasting water in surgical scrub or wasting drugs) that did not directly or indirectly evaluate the environmental impact of the waste, studies that increased productivity for the same resource use but did not quantify the environmental costs per unit activity, and editorials, unstructured narrative review articles, and other publications that did not report any primary data or did not present new analyses of existing data.

Covidence review software (Veritas Health Innovation, Melbourne, Australia) was used for screening. Each title and abstract were screened independently by two reviewers to exclude publications that clearly did not meet the inclusion criteria. Subsequently, potentially relevant full-text articles were retrieved for review. Two reviewers independently assessed each article against the inclusion and exclusion criteria. Any discrepancies between reviewers were resolved by a third reviewer.

### Data extraction

We developed a spreadsheet form for data extraction; the author group tested and reviewed this with example publications. For each publication, we recorded the title, year of publication, study design, country of origin (defined as the country in which the study was located) and World Bank income level of that country, primary outcome measure, means of quantifying environmental impact, statistical analysis of primary outcome, and other results of note. Study details were extracted independently by two authors for all included studies and their results compared. Differences were discussed and resolved, with recourse to other authors in the event of uncertainty. Results were thematically analysed and reported with no opportunity for meta-analysis.

## Results

**Figure 1:**
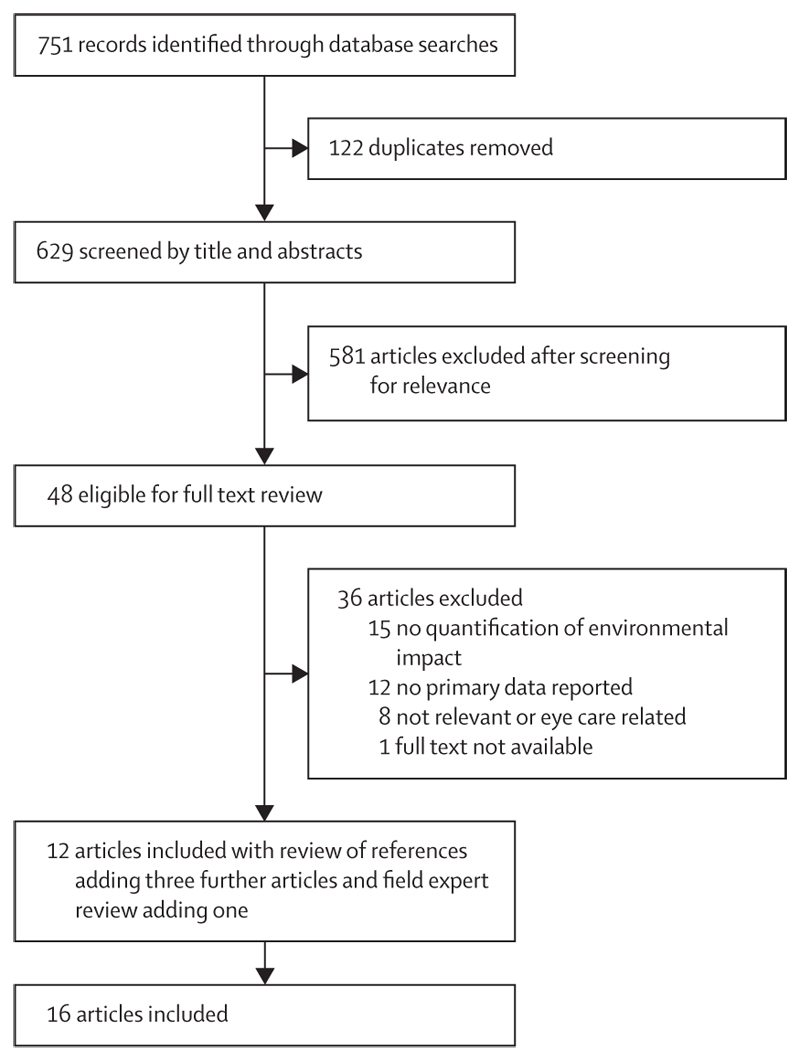
Preferred reporting items for systematic reviews and meta-analyses flow diagram

**Figure 2:**
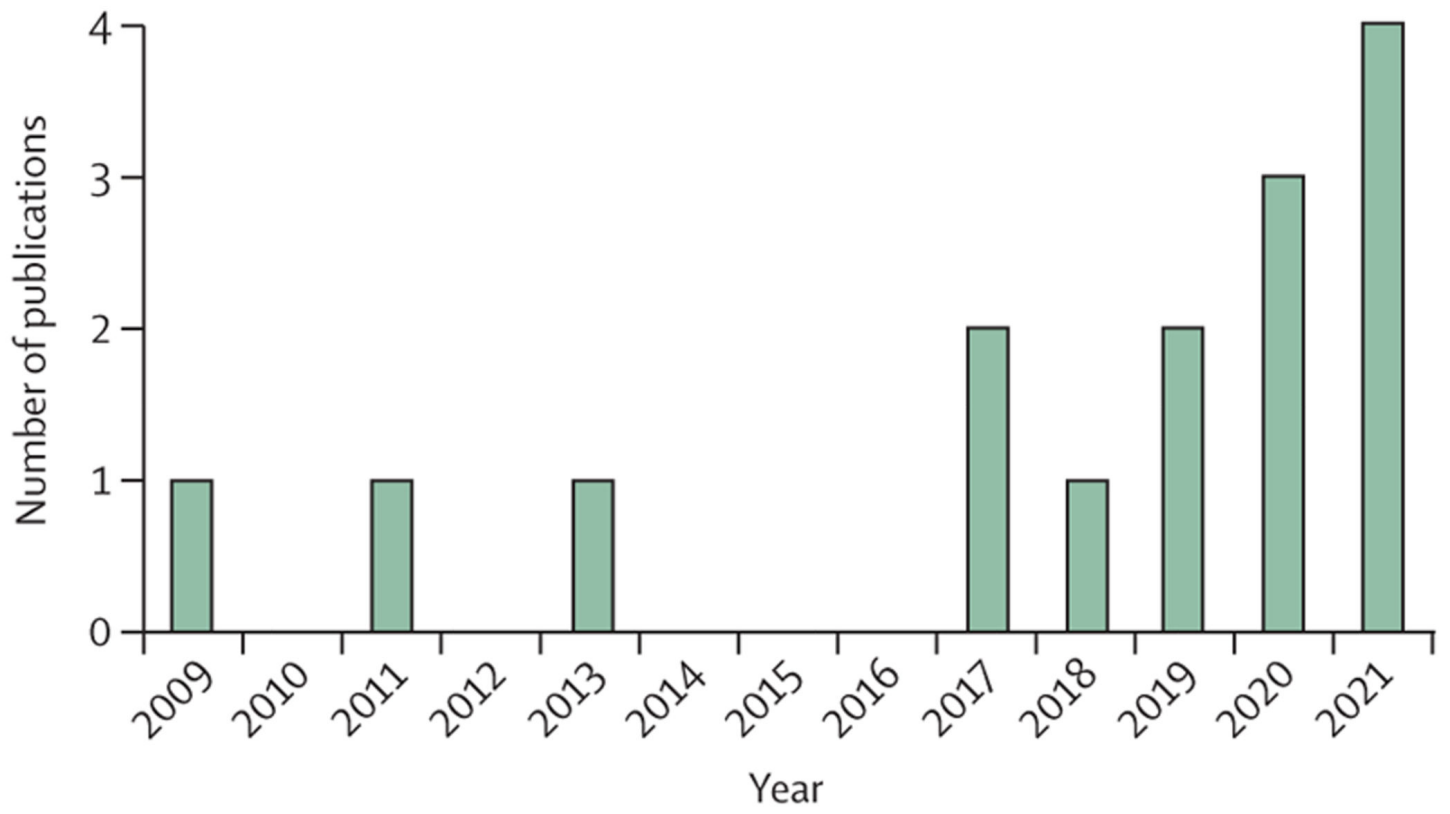
Number of studies meeting the inclusion criteria by year of publication

Our searches returned 751 publications. Removal of duplicates, titles and abstract screening, and further exclusions left 12 articles which met our inclusion criteria. Examination of papers’ reference lists yielded three more studies and the field expert review added one (figure 1).^22–25^ The 16 included studies were all published from 2009 onwards, with an increasing rate of relevant publications in recent years (figure 2). Studies were done in the UK,^26–30^ the USA,^23,31,32^ India,^22,23,33^ New Zealand,^24,34^ Malaysia,^35^ Ireland,^25^ France,^36^ and as international collaborations (table).^38^

### Study designs

One interventional study on paediatric ophthalmic examination was identified.^33^ This study was a randomised controlled trial that compared sevoflurane induction dose only with the standard of care, which is induction with additional maintenance sevoflurane for general anaesthetic. This trial was undertaken from an anaesthesiology perspective. The two groups had equally adequate anaesthetic effect and the induction-dose-only group had a reduction in both financial and environmental costs.^33,39^

One study presented the validation of the Eyefficiency App (version 1.7.3), a tool to enable sustainability audits for cataract surgery.^31^ This tool, and some of the other included studies,^22,24–26,31,32,34–36^ made use of lifecycle assessment, which quantifies the emission of a product or process across its lifecycle (from raw material extraction, to manufacturing, use, and disposal). Lifecycle assessment is conducted using ISO 14040 standards,^40^ and there are two generic approaches. The first is a process-based approach that makes use of quantities of physical inputs to estimate emissions—for example, kilograms of polypropylene plastic. The second is the economic input–output or environmentally extended input–output approach which makes use of financial data in specific economic sectors—for example, the 2013 US$ value of petrochemical plastics. Most health-care lifecycle assessments make use of a hybrid approach, combining process-based and environmentally extended input–output lifecycle assessments. This approach is favoured due to the limitations of existing process-based databases for health-care products such as the absence of data on the environmental costs of pharmaceuticals in lifecycle inventory databases.^41^ The Eyefficiency app and some included studies implement this hybrid approach^22,34,36,38^ and other studies only make use of an economic approach.^26,32^

The other 14 studies included were observational and quantified the environmental impacts of interventions or clinical pathways, in some cases comparing options such as the environmental impacts of different retinal angiography modalities.^27^ Six studies considered cataract surgery^22,26,28,34,36,38^ and two considered intravitreal injections,^24,25^ providing us with estimates of carbon costs from different settings or with different techniques and clinical pathways. Two studies explored pharmaceutical waste in eye care,^29,32^ two detailed the solid waste generated by surgical procedures (one trabeculectomy^23^ and one cataract^35^), and one estimated the environmental impact of the use of sulphur hexafluoride for retinal detachment surgery.^30^

Methodological detail availability was varied, ranging from a published abstract from the Association for Research and Vision in Ophthalmology^27^ to an in-depth component analysis of carbon costs of a cataract pathway that even included estimates of the amount of ink used in printed materials;^26^ however, all studies presented new data and analyses (table).

### Environmental outcomes and quantification

14 (88%) of the included publications reported the environmental cost estimates in terms of CO_2_e (table).^22,26–28,32,33^ Calculation of CO_2_e for components of a service, such as buildings, travel, and procurement, would be impracticable for each team of investigators to undertake; however, carbon footprints can be calculated in a standardised way with publicly available standard carbon cost frameworks,^42^ which allows for comparisons. The remaining two studies did not report any direct measure but reported the weights of waste generated.^23,29^ The benefits of conversion to CO_2_e as a research sector standard is shown by the easy comparability, although the legitimacy of comparison is dependent on equivalence in the assumptions made and methods of conversion. Although aligned on CO_2_e as the unit of outcome, studies were less aligned on what is included in that estimate. For example, three estimates incorporated staff and patient transport,^24,34,36^ but one just included patient transport.^25^ Sufficient disaggregation of the published data allows readers to extract components to compare similar services in different contexts, but other factors still compromise comparability. Greenhouse gas emissions attributed to power consumption in the study of cataract surgery from Wellington, New Zealand (1·8 kg CO_2_e) were very low compared with the same procedure in the UK (66·7 kg CO_2_e).^34^ This disparity was due to the UK study including perioperative visits in their measurement of greenhouse gases, which potentially included power use in the clinics and larger areas of the hospital and the emissions of coal boilers for heating. With less than 50% of UK electricity being derived from renewable energy compared with 82% in New Zealand, UK electricity production produces nearly six times more CO_2_e per unit than does New Zealand’s.^34^

Despite these disparities, attempting to extract the same components for comparison is to some extent possible from the more detailed publications such as that from Morris and colleagues.^26^ This study presented a headline figure of 181·8 kg per cataract case,^26^ but exclusion of items that were not directly comparable resulted in a figure of 130 kg CO_2_e to compare with the 6 kg produced for the same operation in Aravind, India.^22,26^

As with many studies, the authors try to translate those into lay terms to help the reader engage with the CO_2_e estimate; in this example the UK surgery generated a greenhouse gas equivalent to driving a passenger car 500 km, whereas the Indian service equated to driving the same car just 23 km.^22^ Other studies described the area of forest needed to absorb this amount of carbon in one year,^34^ the litres of petrol burned to equal this amount of CO_2_e,^24,34^ or the CO_2_e released by specific international flights.^25^

Only one study^32^ attempted to quantify other environmental outcomes, considering air pollution, which was measured in kg equivalents of fine particulate matter with a particle diameter of less than 10 μm, and the eutrophication potential, which is most frequently reported as the equivalent mass of phosphates, but in this study was reported as mass of nitrogen equivalents, a measure shown to have direct effects on global and individual health.

### Balance of risks

Within included studies, a recurring theme was the tradeoff between the small and unquantified risks to individuals and the population risks from environmental damage. The risk of transmitting infection between patients by reusing eye drops is not known, but is traded against an estimated financial cost of between £2·75 million and £4·6 million per annum for the UK National Health Service and the generation of 6·9–11·4 more tonnes of paper waste and 12·7–21·2 more tonnes of plastic waste, without any consideration of whether this trade represents good value.^29^ Similarly, in surgery, the risks of contamination or clustering of adverse reactions or infections that are avoided by discarding unused medications comes at a financial and environmental cost.^32^ The same point has been made regarding single use tonometer prisms (for measuring intraocular pressure) and gonioscopy lenses.^43^ An unquantified theoretical risk of transmitting corneal infections between patients is theoretically reduced to zero at a quantifiable financial and environmental cost. Even the apparent convenience of disposable items is not sufficiently questioned, as there are hidden inconveniences and costs in the work to procure, stock check, store, and dispose of packaging, which are largely avoided with reuse.

Optical coherence tomography angiography carries no clinical risks and has lower environmental costs than traditional fundus fluorescein angiography and can be done in its place for evaluating retinal vascular changes in about half of patients.^27^ The remaining patients will still require fundus fluorescein angiography due to their specific indications or comorbidites. The opportunity cost of capital expenditure on optical coherence tomography angiography, diverting funds from other health-care activities, needs to be weighed against the clinical risk and environmental damage that would be avoided. This same dynamic is present for any innovation aimed at incrementally improving eye health; the potential benefits need to be proportionate to the environmental costs.

### Recycling

A Malaysian study^35^ focusing on cataract surgery waste generation found that segregation of waste and recycling could reduce total waste-related emissions from 0·421 kg CO_2_e per phacoemulsification to 0·282 kg CO_2_e. Although this change could cause a 30% reduction in carbon footprint, 0·139 kg CO_2_e saving per case is not revolutionary, as it is less than 1% of the total carbon costs of a cataract case. Another example of the secondary importance of recycling came from the New Zealand study of cataract surgery,^34^ in which hospitals that recycled still produced more unrecyclable solid waste than those that did not recycle. A previous report suggested that behaviour change might need to be introduced into training programmes, because trainee surgeons generate almost a quarter more waste than experienced surgeons, an excess usage that could be amenable to improvement by education.^35^

### Procedure-specific sustainability priorities

In HICs, intravitreal injections have become the most common ophthalmic procedure, meaning that, although the estimated CO_2_e per intravitreal injection is much less than cataract surgery in absolute terms, intravitreal injections are still a large and growing part of the overall eye care carbon footprint.^25^ Papers from HICs suggest that those wishing to reduce the emissions from cataract surgery should aim to increase the numbers of operations per operating list. This increase would spread the fixed costs of buildings and staff transport across more operations so that the per operation carbon costs are reduced. This change complements the targeting of procurement costs, which constitute the majority of emissions.^26,34,38^ Estimates for intravitreal injection identify patient transport costs as the most important factor, representing 40–77% of the non-pharmaceutical footprint, hence sustainability interventions might target longer-acting medications that reduce frequency of visits, sustainable transport options, or services closer to home.^24,25^

### Other publications

Of the 36 papers excluded during full-text review, 27 related thematically to environmental sustainability in eye care, but did not report environmental emissions or include primary data. Many were editorials discussing sustainability.^44–49^ Some explored sustainability-related topics without quantifying emissions, such as investigations of the cost and quality of reusable and single-use ophthalmic instruments^43,50–52^ or practices of recycling in eye care.^53–55^ One study assessed the opinions of New Zealand ophthalmologists regarding sustainability.^56^ The survey showed that surgeons are interested in making more sustainable changes that might also decrease costs and increase value of eye care, although 19% (9/47) expressed the opinion that climate change was not driven by human behaviour and did not need mitigation.^56^ Similarly, a more recent online survey of 1300 US ophthalmologists and nurses reported that 93% believed that operating waste is excessive and should be reduced, 90% were worried about global warming, but only 78% wanted to reuse more supplies—possibly reflecting the difficulty of turning non-specific environmental concerns into specific changes in practice.^57^

## Discussion

The belief that human activity, primarily mediated through greenhouse gas emissions, is the major driver of climate change is generally acccepted.^58^ The substantial contribution of health care to greenhouse gas emissions has also been established.^8^ Therefore, it is striking how little data are available from the eye-care sector that quantify the environmental impacts of services, and how few studies exist that design, trial, and implement interventions to reduce these impacts. The single interventional study in this Review,^33^ although conducted in an ophthalmic theatre, is really an anaesthetic study. In reference to the direct greenhouse gas effect of anaesthetic inhalation agents, Datta and colleagues^33^ explain that the “major limiting factors” for routine use of sevoflurane are the drug’s high cost and its associated environmental effects. Environmental considerations have not yet, to our knowledge, been referred to as a major limiting factor to an intervention or technology in any publication about eye care. Some eye-care professionals have engaged with environmental issues in the broader health-care arena, such as a study comparing different surgical scrubbing arrangements and their environmental impacts;^39^ however, no specific interventional study about eye care was identified in this Review.

It is not clear what impact this paucity of evidence from the eye-care research sector has had on policy makers, clinicians, and researchers. Wider reading provides examples in which evidence-based behaviour changes to promote environmental sustainability have occurred in other medical specialities.^59^ For example, when the environmental benefits of asthma inhaler devices that did not make use of greenhouse gas propellants was quantified,^60^ environmental impact became one of the factors patients were offered to consider when deciding which type of inhaler to use.^61^ There are, however, no examples of strong evidence-based advocacy leading to changes in policy environments to allow the adoption of sustainable practices in settings which currently mandate unsustainable practices. Nonetheless, it must be assumed that such policy change, and resultant behavioural changes, are less likely in the absence of a robust evidence base. The evidence around anaesthetic gases used in ophthalmic theatres might have increased clinician motivation to reduce the use of these powerful greenhouse gases in ophthalmology.^62^

However, there are signs that this research agenda is growing. Despite not using a publication-date exclusion criteria, the articles included in this Review were all published after 2009, and eight of the 16 publications date from 2020 or later (figure 2). This finding is consistent with the perception that the serious nature of the environmental impact of health care has only recently been acknowledged.^2^

**Figure 3:**
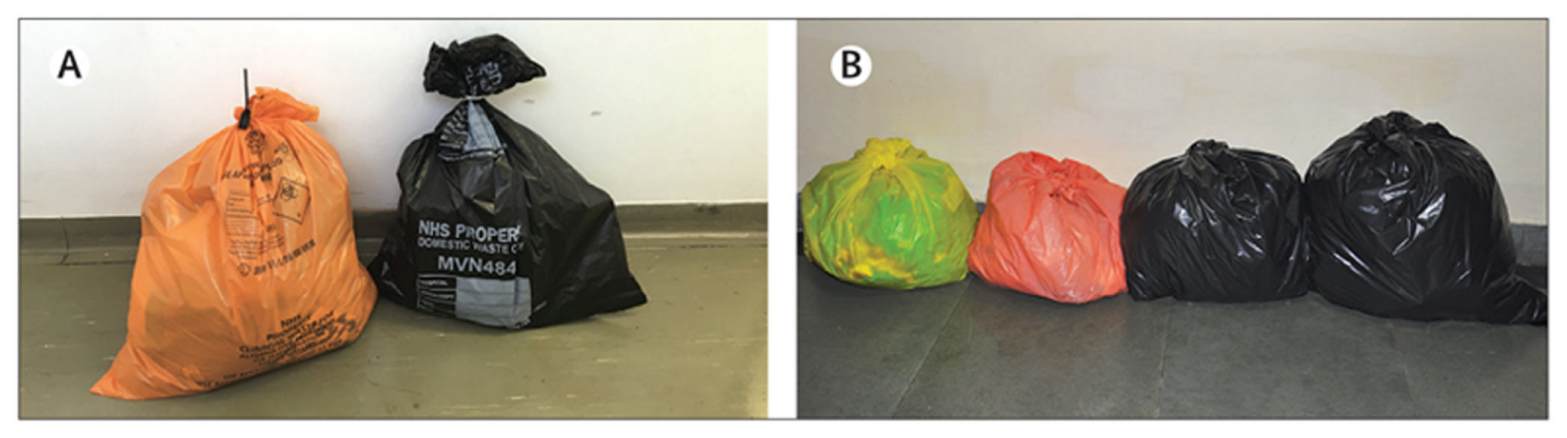
Waste produced from one phacoemulsification in the UK (A) and 32 cases in India (B)

Beyond the low engagement with the issue of climate change by the international research community, the other finding of this Review is the difference between the carbon footprint of similar services delivered in different settings: one phacoemulsification in the UK produces more than 20 times the greenhouse gas emissions of one phacoemulsification in India, with both having excellent clinical outcomes.^22,26,63^ This contrast is also portrayed visually in images taken for *The Lancet Global Health Commission on Global Eye Health: vision beyond 2020*,^20^ which showed the waste generated for one phacoemulsification procedure in the UK and the waste produced for 32 phacoemulsifications in India (figure 3).

Where comparable outcomes can be delivered at reduced environmental costs,^49,63^ an opportunity exists for the migration of practice; convenient lifecycle assessment tools such as the Eyefficiency App permit the evaluation and cyclical audit of the carbon footprint of cataract surgical services.^31,38^ However, authors also attribute the large difference between the Indian and UK cataract services to the policies in HIC settings that prevent the adoption of sustainable practices. Because of these policies, full realisation of the potential gains will require changes to the regulatory environment^22^ and a change of culture, such that any incremental improvements suggested for safety or better outcomes (eg, the introduction of laser-assisted cataract surgery), be measured against both the financial and environmental costs required to achieve those gains.

### The case for cultural and behavioural change in highincome settings

The assertion that current practice in HICs is unsustainable needs justification. As an example, a calculation of the carbon footprint of cataract surgery worldwide can be made and extrapolated to the anticipated need for cataract surgery after the predicted stabilisation of the world population at about 10 billion people.^64^ If the aspiration of universal health coverage as part of SDG3^15^ is to be realised, then health-care economies of the world must be expected to match their delivery of cataract surgery to rates typically found in HICs (8000 cataract operations per million population per year) as LMIC populations grow and age with increasing life expectancy. Considering the estimate of 181·8 kg CO_2_e per cataract surgery in the UK,^26^ at a world population of 10 billion, global cataract surgery would generate 14·5 Mt of CO_2_e annually. To put this into context, if the world moved to current UK exemplar practice, then at a world population of 10 billion, cataract surgery alone would create similar annual greenhouse gas emissions to entire countries such as Kenya (14·3Mt), Croatia (16·8Mt), or Sri Lanka (18·4Mt; 2014 World Bank CO_2_e estimates), which is not sustainable. Some reduction in that total could be possible working within current policies. However, to realise the goal of carbon neutral economies, changes in HIC policy environments will be needed to permit cataract surgical providers to adopt practices similar to those in LMIC settings that have been shown to be clinically safe,^63,65^ and protect population and planetary health by protecting finite environmental resources.

### Balance of risks

Across the papers there was a discussion about a trade off between the potential risks to individual patients and the risks to the population as a whole; this discussion is seen across health care. In the UK, the risk of transmission of Creutzfeldt-Jacob disease led to an increase in use of single-use surgical instrumentation. This change resulted in an unknown extent of avoidance of transmission; it is possible that no benefit has been gained from this shift in practice.^66^ By contrast, the additional financial and environmental costs of transition to disposable surgical instruments are clear and quantifiable. If the total amount of public money for health care is assumed to be a fixed amount, the money spent on disposable instruments is therefore not available to convert to quality adjusted life-years (QALY) elsewhere in the system. However, the patients who lost those QALYs due to the money spent on disposable instruments cannot be individually identified.

We suggest that changes in thinking regarding patient safety are needed. New medical interventions are required to show a certain degree of cost–utility before health-care funders will agree to support their introduction. No such cost–utility threshold is set for interventions or policies claiming to increase safety, therefore practices that offer very poor returns on investment can be promoted or even mandated; for example, prion-removing blood filters to prevent Creutzfeldt-Jacob Disease transmission from transfusions were estimated to cost €3·7 million per QALY.^67^ Similarly, no consideration is given to what mass of CO_2_e per QALY gained is acceptable. There is no clear correlation coefficient to convert environmental damage to loss of QALYs at the population level that could be factored into cost–utility analyses, but it would seem intuitive that there should be a carbon cost per QALY that is unacceptable, both for interventions that claim to promote safety and for those that offer improved outcomes, just as there are financial costs per QALY thresholds.

We identified a study of the risk of creating a potential transmission of bacteria by reusing eye drops.^29^ If reuse was permitted, then a defined saving of resources, that can be quantified financially and environmentally, could be diverted to purchase QALYs elsewhere in the system and to protect the environment. This opportunity is lost in reducing the risk of transmission of microbes between patients to zero. Quantifying the actual clinical risk of using multidose eye drops for sequential patients is problematic. Videographic analyses and bacterial cultures can be used to estimate the level of risk,^29,68^ but in a real-world setting, clinicians who are reusing eye drops for multiple patients would exercise judgement regarding which patients they felt unsafe to reuse drops from (such as emergency eye-care settings with infective conjunctivitis patients) and which patients had an acceptably low risk of pathogen transmission. Because we cannot quantify the risk of reusing eyedrops, it is unclear whether we are overpaying for a reduction of certain risks to zero, when we are taking on other risks due to greenhouse gas emissions, the negative impacts of which will most acutely be felt by people in LMICs in the future.^69^ This creates intergenerational and international inequality, although the impact of climate change is already evident in LMICs as detailed in the *Lancet* countdown on health and climate change annual report.^70^ Most of the authors from the reports identified in this Review are from HICs, which is consistent with the reality that the majority of greenhouse gas emissions are produced by HICs, but it has been estimated that 92% of pollution-related deaths occur in LMICs.^69^

Some hope can be derived from examples where, with strong policies and procedural approaches, multidose bottle use has been deemed acceptable, even in a high-income and highly litigious environment such as the USA.^71^ The American Society for Dermatologic Surgery support the freezing and repeated use of multidose botulinum toxin^72^ and the American Academy of Ophthalmology has published recommendations regarding safe use of multidose drugs in the perioperative setting,^73^ but it is unclear whether such recommendations translate either into change of practice or medicolegal protection for those following them.

### Future directions

**Figure 4:**
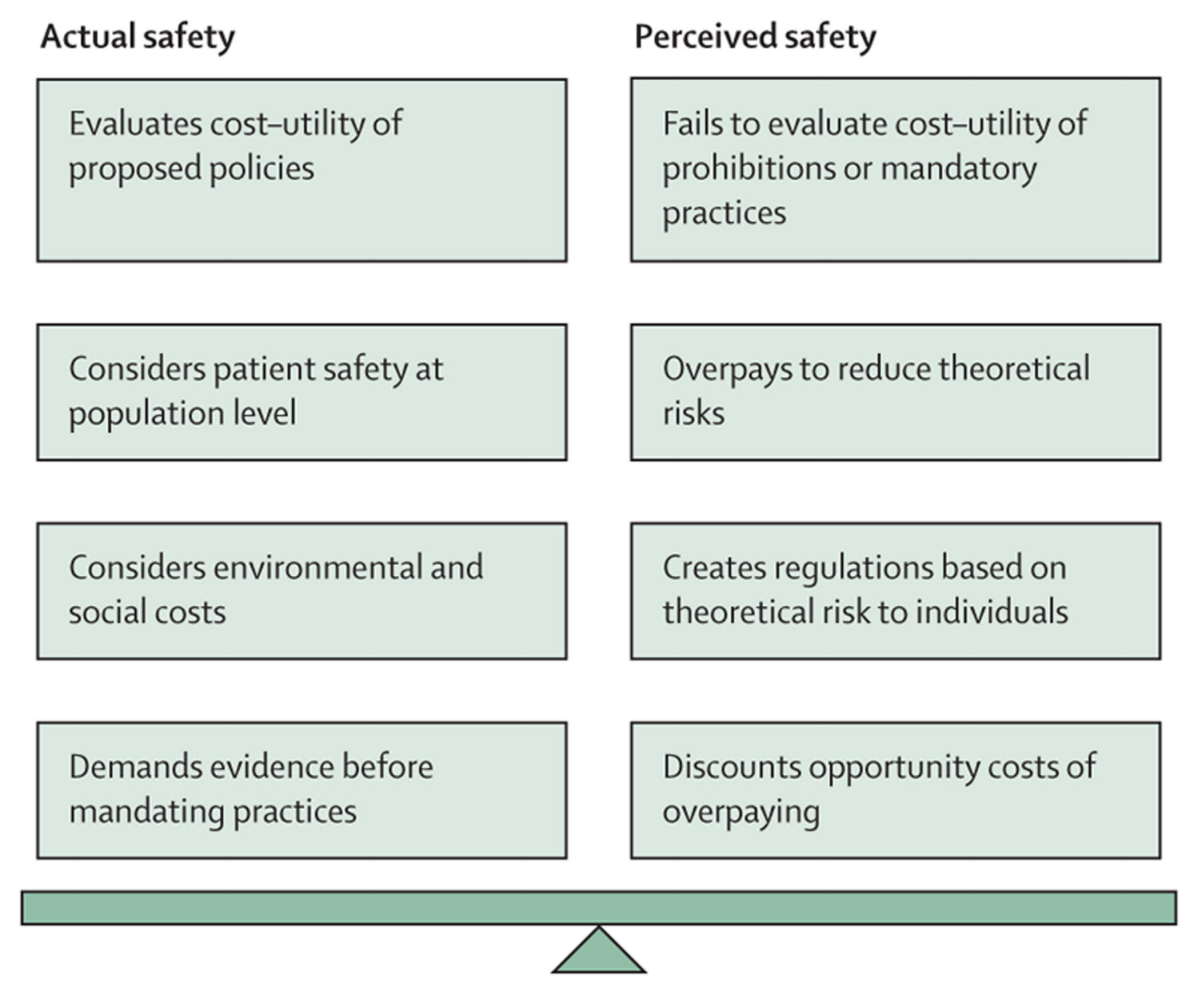
Balance of risks between actual and perceived safety promoted by health-care interventions

When an ophthalmologist is considering treatment options for a patient, some believe that “global environmental issues are mainly irrelevant at that acute point in time”.^19^ Certainly, it could be argued that the individual clinician is constrained to consider the best interests of the individual patient in front of them without consideration of the wider societal, financial, or environmental costs. However, as health-care professionals, managers, and academics, we have an equally strong duty to the collective of patients (within which we are a subset), to configure our health-care service delivery in a way that protects the public health interest—an interest that is inextricable from the interests of the physical environment we all live in. If the environmental costs and the opportunity costs of redirected finances were fully considered, it is likely that some interventions aimed at promoting safety, would be found to result in a net harm. Therefore, we wish to contrast those interventions that promote patient safety in actuality with those that create only the perception of safety (figure 4). This recurring theme in the papers calls us to ask whether we can dismantle an entrenched notion of safety that has no evidence base.

It could be that researchers from less well-resourced health-care systems have a pivotal role to play in designing and trialling sustainable interventions in eye health-care delivery. There are examples of frugal innovation in which LMIC health-care practices could drive resource-efficient practice in HICs,^74^ and the randomised controlled trial identified in this Review is an example of this.^33^ Researchers in HICs could struggle to obtain ethical committee approval to trial sustainable interventions that contradict existing policies. Real world evidence of the safety profile of, for instance, reusing eye drops (such as dilating drops in routine diabetic retinopathy screening services) might need to come from LMIC settings.

Evidence alone might not be enough unless policy makers in HICs can tackle their unconscious biases. Regardless of how safe the practice of reusing gloves for multiple patients is (with interoperative disinfection of the gloves), the regulatory frameworks in HICs would prevent trialling or adoption of this practice.^75^ Even the practice of autoclaving unwrapped instrument sets between operations in theatre, which was historically common practice, is now frequently prohibited in cataract surgical services in HICs despite not being shown to be associated with any increased risk of patient harm compared with more energy intensive sterilisation options.^76^

A truly evidence-based approach to clinical safety that considers environmental impacts is clearly necessary if we are to move to a position in global health where HICs do not enforce practices that would be unsustainable if done globally.

## Conclusion

This Review found only 16 studies that estimated the environmental footprint of eye-care services, only one of which trialled an intervention to diminish environmental damage. There is an urgent need for more research in this area, which should include quantifying emissions from a broader range of eye-care services and activities and expanding analyses to include environmental consequences beyond greenhouse gas emissions. Routine inclusion of environmental outcomes in clinical trials should be an aspiration and the specific identification, testing, and promotion of environmentally-sustainable practices. The buying power of a large speciality such as ophthalmology gives it the opportunity to have an impact across the health sector, encouraging sustainable manufacturing, packaging, and energy supplies.

If the global health community is indeed a community, then evidence gaps identified in HICs might need filling by LMIC colleagues showing that safe and high-quality outcomes are achievable with practices that can be sustainably scaled up to meet the needs of the ageing and growing global population. It is contrary to the global public health interest to have a small group of HICs enforcing unsustainable practices which have climate-related repercussions that are felt predominantly by the health systems least well-resourced to deal with them. All human activities must lie within our planetary boundaries.

## Supplementary Material

Supplementary Material

## Figures and Tables

**Table 1:** Summary of the 16 included studies investigating environmental sustainability in eye care

	Country, year; study design	Study design summary	Results	Environmental impact (units)
Ophthalmology carbon footprint: something to be considered?^28^	UK, 2009; observational	A short report estimating GHG emissions of phacoemulsification compared with MSICS; one-stop cataract pathway compared with a five-stop pathway with hospital-based follow-up	Lens extraction with phacoemulsification resulted in an excess of 280 g of plastic waste, 8 g of paper waste, and 78·7 g of CO_2_e compared with MSICS; the difference in CO_2_ emissions between a 5-stop and a 1-stop cataract pathway was 29·8 kg of CO_2_e per case due to travel	CO_2_e
The precautionary principle: what is the risk of reusing disposable drops in routine ophthalmology consultations and what are the costs of reducing this risk to zero?^29^	UK, 2010; observational	Single-use eye drops (n=100) were sent for agar plate culture after use for one patient each; paper and plastic waste was weighed; an estimate of the number of eye drops instilled in UK NHS eye services was calculated and total annual potential savings of waste and money from reuse of drops estimated	5% of single use droppers cultured coagulase negative staphylococcus; reuse of each unit just once would save the UK NHS annually £2·75 million, 6·85 tonnes of paper waste, and 12·7 tonnes of plastic waste, but with an estimated 31 730 potential cross-contamination events; the clinical implications of transmission of pathogenic or commensal bacteria by reusing eye drops are unquantified, but health-care providers pay to reduce this risk to zero; the financial and environmental resources used to reduce this risk could be redirected to provide other services	Mass of waste
The carbon footprint of cataract surgery^26^	UK, 2014; observational	Component analysis study including both direct and indirect GHG emissions from activity attributable to an individual patient undergoing first eye cataract surgery from referral to discharge; emissions were collated from building and energy use, travel of patients and staff, and procurement of surgical consumables	One phacoemulsification cataract operation in the UK generated 181·8 kg CO_2_e; breakdown by sector showed emission contributions: building and energy use 36·1%, travel of patients and staff 10·1%, procurement of surgical consumables 53·8%; detailed component analysis permits other centres doing similar studies to make valid comparisons	CO_2_e
Anaesthesia maintenance with ‘induction dose only· sevoflurane during paediatric ophthalmic exam: comparison with standard low flow technique RCT^33^	India, 2016; interventional	RCT in paediatric ophthalmology operating theatres randomly assigning 50 children undergoing examination under anaesthesia to different general anaesthetic strategies; all children were induced with 8% sevoflurane in O_2_:N_2_O (40:60); standard regime of 2% sevoflurane at 1L/min fresh gas flow O_2_W_2_O (50:50) was compared with 0·5 L/min fresh gas flow O_2_M_2_O (50:50) without any sevoflurane	General anaesthetic was maintained equally in each group (median examination time 14-15 min); the induction dose only group used 2 mL less sevoflurane per case and 3·75 mL less nitrous oxide per case; if this anaesthetic strategy was used researchers estimate this will reduce CO_2_e by 11 327 L per theatre day which converts to 22·6 kg CO_2_e; this study was conducted by anaesthetists, and just happened to be in an ophthalmology theatre; as such it is indicative of the attention environmental issues are receiving in other specialities	CO_2_e
Cataract surgery and environmental sustainability: waste and lifecycle assessment of phacoemulsification at a private healthcare facility^22^	India, 2017; observational	Evaluation of the environmental impact of cataract surgery in two large south Indian centres; they compared their emissions with relevant components from the UK study^34^	One phacoemulsification in an Indian centre generated 0·25 kg of waste and 6 kg CO_2_e; the CO_2_e from comparable components of the pathway is around 5% of that produced per case in the UK setting	CO_2_e
Waste generated during glaucoma surgery: a comparison of two global facilities^23^	India and USA, 2018;observational	The amount of waste per trabeculectomy is estimated from a centre in Baltimore, MD, USA and a centre in Madurai, India; standard conversions for waste can be applied to quantify different GHG emissions	Mean waste per trabeculectomy was 0·5 kg (range 0-3-0-7; p>0·5) in Aravind, India, compared with 1·4 kg per trabeculectomy (range 1-0-1-8; p>0·05) in Baltimore-area hospitals, USA; the authors explain the difference is because “certain regulations in the US lead to the production of potentially unnecessary waste”	Mass of waste
Quantification of the cost and potential environmental effects of unused pharmaceutical products in cataract surgery^32^	USA, 2019; observational	Quantified the financial and environmental impact of practices in four cataract surgical centres in the USA from January, 2016, to February, 2018	Across the four centres, a mean 45·3% of pharmaceuticals were unused (range 16·0-60·2%); the financial and environmental implications of unused medication are presented for each facility compared with full utilisation of medication; the excess CO_2_e ranged from 418 kg/month to 2498 kg/month; excess air pollution ranged from 0·8 kg PM_10_-e/month to 4·5 kg PM_10_-e/month, eutrophication potential from 0·07 kg N-e/month to 0·42 kg N-e/month; across the four centres, per cataract case, unused medication results in 6-30 kg CO_2_e excess emission compared with full use, at a cost of US$41-217 per cataract case	CO_2_e, PM10-e, and kg N-e
The carbon footprint of fluorescein angiography compared to OCT angiography^27^	UK, 2019; observational	OCTA is an alternative to FFA that can be used in around half of cases; the environmental benefits of converting to OCTA are presented; in some settings OCTA can be performed immediately in clinic but FFA might require a subsequent visit; separate estimates are generated assuming FFA requires a separate visit or is available on the same day	The additional GHG associated with FFA compared with OCT angiography per patient are building costs of 40·17 kg CO_2_e, travel of staff of 33·03 kg CO_2_e, patient travel of 4·27 kg CO_2_e, pharmaceuticals of 1·01 kg CO_2_e, medical instrumentation of 1·41 kg CO_2_e, and waste of 1·24 kg CO_2_e; in total, FFA creates an excess of 76·24 kg CO_2_e per patient if done on the same day as their outpatient appointment, and 80·51 kg CO_2_e per patient if done at a subsequent appointment; if 50% of FFA were converted to OCTA, this would save 34 tonnes CO_2_e per year in one UK hospital currently doing 600 FFA per year	CO_2_e
(Continued from previous page) Waste production from phaco surgery^35^	Malaysia,2020 observational	Prospective study of waste from all phacoemulsification- only operating lists over 6 months in 2017 categorised into general waste (ie, paper and packaging), clinical waste, and sharps and medication	Average waste production 0·827 kg per phacoemulsification; experienced ophthalmologists averaged 0·814 kg, trainees averaged 1·086 kg; only 0·159 kg (51%) of general waste was recyclable; using GHG conversion factors this waste is estimated as 0·282 kg CO_2_e per phacoemulsification if optimising recycling, increasing to 0·421 kg CO_2_e without segregation	Mass of waste converted to CO_2_e by standard factors^37^
Utilizing off-the-shelf LCA methods to develop a ‘triple bottom line’ auditing tool for global cataract surgical services^31^	USA, 2020; description of application development	The Eyefficiency (version 1.7.3) tool, an audit app to permit cyclical evaluation of efficiency (financial and environmental costs) for cataract surgery, is validated using published detailed financial and environmental costs of cataract surgery from two of Eyefficiency’s pilot sites; three different LCA approaches; GHG emission estimates from the costs of surgery and the carbon equivalent results are compared with previous studies^22,34^	Accuracy in terms of approximation to the original studies GHG emission estimates was poor, with the UK site estimate being 76% of the original and the Indian centres’ estimate being 476% of their original; the benefits of adopting an easily updated and convenient LCA approach outweigh the need for absolute accuracy, because the predominant use is internal quality improvement cycles; comparison between contexts is still possible as the differences between GHG emissions from HICs and LMICs is currently so large (20-fold difference) and is greatly in excess of the degree of any inaccuracy introduced by the LCA method	CO_2_e
Response to Tetsumoto et al. The environmental impact of fluorinated gases^30^	UK, 2020; observational	Responding to an article showing that air works equally to SF6 in retinal detachment surgery, authors show that SF6 is the most potent greenhouse gas regulated by Kyoto protocol (global warming potential 22 800 x CO_2_); annual usage estimates of SF_6_/C_2_F_6_ for their unit are presented and CO_2_e calculated	6·48 L of SF_6_ and 12·24 L of C_2_F_6_ equates to 2·7 tonnes of CO_2_ for their hospital eye department annually	CO_2_e
Improving productivity, costs and environmental impact in international eye health services: using the ‘Eyefficiency’ cataract surgical services auditing tool to assess the value of cataract surgical services^38^	USA, Mexico, Chile, Swaziland, South Africa, India, New Zealand, Hungary, UK, 2021; observational	Nine international cataract surgical facilities used the Eyefficiency app (version 1.7.3) to collect data (staffing, pathway steps, costs of supplies, energy use, and live time-and-motion data) for 1 week or 30 consecutive cataract surgeries; Eyefficiency quantifies productivity, costs, and carbon footprint	Nine sites recorded 475 cataracts (mixture of phacoemulsification and manual small incisions); operations per hour ranged from 1·47 to 4·48. Average per-case expenditures ranged between £31·55 and £399·34, with a majority of costs attributable to medical equipment and supplies; average solid waste ranged between 0·19 kg and 4·27 kg per phacoemulsification, and CO_2_e from 41 kg to 130 kg per phacoemulsification	Solid waste mass and CO_2_e
The carbon footprint of cataract surgery in Wellington^34^	New Zealand, 2021; observational	Carbon footprint of phacoemulsification surgery was estimated from up to 40 operations in each of four different hospitals, broken down into energy supply, travel for patients and staff, procurement of disposable items and pharmaceuticals, and waste disposal	The average footprint of cataract surgery was 151·9 kg CO_2_e divided into 1·2% electricity, 15·0% transport, 76·7% from necessary materials, 6·9% pharmaceutical procurement, and 0·13% waste disposal; comparison was made between private hospitals with higher volumes of cataract surgery, and public hospitals with lower volumes, travel emissions per operation were lower in private hospitals, primarily due to longer lists of cataract operations (ie, less staff travel per case); two of the four hospitals recycled, however, despite recycling, those two hospitals still produced a greater mass of solid waste than the others due to higher average waste production	CO_2_e
Analyzing the carbon footprint of an intravitreal injection^25^	Ireland, 2021; observational	Costs associated with IVI were captured at a single unit in Dublin over 3 months; CO_2_e calculated using hybrid LCA method considering: transport of patients, necessary materials, building energy consumption	Carbon emissions associated with a single intravitreal injection, excluding the anti-VEGF agent, were 13·68 kg CO_2_e; this equates to 82 100 kg CO_2_e annually for this IVI service, which divides up to 77% patient travel, 19% necessary materials, and 4% building energy usage; longer acting drugs which reduce visits offer the greatest opportunity for reduced environmental impact of IVI services	CO_2_e
The carbon footprint of intravitreal injections^24^	New Zealand, 2022; observational	Estimated the carbon footprint of 226 IVI at four public injection-only clinics in the Wellington region and quantified the disposable materials used by injectors across New Zealand; carbon costs were included from travel of both staff and patients, building energy use, waste disposal, and pharmaceuticals	The disposable materials, travel, building energy, and waste disposal had a combined emissions footprint of 14·1 kg CO_2_e, equivalent to a 6 L petrol burn, or driving 75 km; the injected medication itself had a much greater estimated carbon footprint than the other components but varied greatly by agent used (bevacizumab 16·5 kg CO_2_e, aflibercept 375 kg CO_2_e)	CO_2_e
The carbon footprint of cataract surgery in a French university hospital^36^	France, 2022; observational	Data collected from a single day of operating on 12 cataracts in one hospital in Paris; broken down as transport of both patients and staff, building energy use, and procurement of pharmaceuticals and disposable medical devices	Average CO_2_e for one phacoemulsification was 81·13 kg; this is broken down into transport 9·0%, energy 3·4%, procurement of disposable medical devices 73·3%, procurement of pharmaceuticals 12·7%, and waste disposal 1·4%	CO_2_e

CO_2_e=carbon dioxide equivalents. FFA=fundal fluorescein angiogram. GHG=greenhouse gases. HIC=high-income country. IVI=intravitreal injection. LCA=lifecycle assessment. LMIC=low-income and middle-income country. MSICS=modified small-incision cataract surgery. N-e=nitrogen equivalents. NHS=National Health Service. OCT=optical coherence tomography. OCTA=optical coherence tomography angiography. PM_10_-e=particular matter <10 μm equivalent. RCT=randomised controlled trial. SF6=sulphur hexafluoride. VEGF=vascular endothelial growth factor.

## Key messages

- A small body of work has emerged since 2009 focusing on the environmental impact of eye care
- Clinical services with similar safety profiles and outcomes have very different environmental consequences depending on the regulatory frameworks in which they exist
- When designing policies and services, we suggest that thresholds should be applied to the amount of environmental damage that is acceptable per quality-adjusted life year gained
