# Supplementary material for: Addressing the environmental sustainability of eye health-care delivery: a scoping review
Source: Lancet Planet Health. Author manuscript; Available in PMC 2025 Oct 23. (PMC7618290; doi:10.1016/S2542-5196(22)00074-2)
Supplement: Supplementary Material [file EMS209638-supplement-Supplementary_Material.pdf]

THE LANCET
Planetary Health

Supplementary appendix

This appendix formed part of the original submission and has been peer reviewed. We post it as supplied by the authors.

Supplement to: Buchan JC, Thiel CL, Steyn A, et al. Addressing the environmental sustainability of eye health-care delivery: a scoping review. *Lancet Planet Health* 2022; **6:** e524–34.

This online publication has been corrected. The corrected version first appeared at thelancet.com/planetary-health on Aug 3, 2022

Appendix 1: MEDLINE, Embase and Global Health search terms

MEDLINE

1. exp Eye Diseases/

2. Ophthalmology/

3. Ophthalmologic Surgical Procedures/

4. exp Cataract Extraction/

5. Lens Implantation, Intraocular/

6. (cataract or cataracts).tw.

7. or/1-6

8. exp Climate Change/

9. Greenhouse Effect/

10. Greenhouse Gases/

11. Carbon Footprint/

12. "Conservation of Natural Resources"/

13. "Conservation of Energy Resources"/

14. "Conservation of Water Resources"/

15. Water Supply/

16. "Environmental Restoration and Remediation"/

17. Green Chemistry Technology/

18. Environmental Pollution/

19. exp Waste Products/

20. exp Water Pollution/

21. (climate adj2 change$).tw.

22. (planetary adj2 health).tw.

23. (environment$ adj2 (sustainab$ or emission$ or impact$ or cost$ or burden$ or wast$ or resource$ or conserve$)).tw.

24. (environment$ adj4 (manufactur$ or product$ or equipment or scrub$)).tw.

25. (unused adj3 (product$ or instruments or equipment or material$)).tw.

26. ((steriliza$ or reusable) adj5 (instruments or equipment$ or material$)).tw.

27. ("single use" adj5 (instruments or equipment or material$)).tw.

28. (waste adj3 (generat$ or audit)).tw.

29. (water adj2 (reduc$ or wast$ or modif$)).tw.

30. (carbon adj4 (emission$ or footprint$)).tw.

31. emissions.tw.

32. (greenhouse adj3 (gas$ or emission$ or effect$)).tw.

33. (global adj2 resource).tw.

34. or/8-33

35. 7 and 34

36. exp animals/

37. exp humans/

38. 36 not (36 and 37)

39. 35 not 38

40. (animal$ or rabbit$ or rat or rats or mouse or mice or chicken$ or dog or dogs or cat or cats or feline or pig$ or monke$ or fish).tw.

41. 39 not 40

42. exp case reports/

43. (case adj2 (report$ or series)).tw.

44. 42 or 43

45. 41 not 44

46. (spectral or photon or otoacoustic or fluorescence).tw.

47. (cell$ or mutation$ or genes or genome or sequencing).tw.

48. 46 or 47

49. 45 not 48

Embase

1. exp eye disease/di, dt, pc, rt, rh, su, th [Diagnosis, Drug Therapy, Prevention, Radiotherapy, Rehabilitation, Surgery, Therapy]

2. ophthalmology/

3. eye surgery/

4. cataract extraction/

5. lens implantation/

6. (cataract or cataracts).tw.

7. or/1-6

8. climate change/

9. greenhouse effect/

10. greenhouse gas/

11. environmental impact assessment/

12. life cycle assessment/

13. carbon footprint/

14. environmental protection/

15. energy conservation/

16. water conservation/

17. green chemistry/

18. pollution/

19. chemical waste/

20. hazardous waste/

21. hospital waste/

22. waste disposal/

23. (climate adj2 change$).tw.

24. (planetary adj2 health).tw.

25. (environment$ adj2 (sustainab$ or emission$ or impact$ or cost$ or burden$ or wast$ or resource$ or conserve$)).tw.

26. (environment$ adj4 (manufactur$ or product$ or equipment or scrub$)).tw.

27. (unused adj3 (product$ or instruments or equipment or material$)).tw.

28. ((steriliza$ or reusable) adj5 (instruments or equipment$ or material$)).tw.

29. ("single use" adj5 (instruments or equipment or material$)).tw.

30. (waste adj3 (generat$ or audit)).tw.

31. (water adj2 (reduc$ or wast$ or modif$)).tw.

32. (carbon adj4 (emission$ or footprint$)).tw.

33. emissions.tw.

34. (greenhouse adj3 (gas$ or emission$ or effect$)).tw.

35. (global adj2 resource).tw.

36. or/8-35

37. 7 and 36

38. (animal$ or rabbit$ or rat or rats or mouse or mice or chicken$ or dog or dogs or cat or cats or feline or pig$ or monke$ or fish).tw.

39. 37 not 38

40. exp case report/

41. (case adj2 (report$ or series)).tw.

42. 40 or 41

43. 39 not 42

44. (spectral or photon or otoacoustic or fluorescence).tw.

45. (cell$ or mutation$ or genes or genome or sequencing).tw.

46. 44 or 45

47. 43 not 46

Global Health

1. exp eye diseases/

2. (cataract or cataracts).tw.

3. or/1-2

4. climate change/

5. greenhouse effect/

6. greenhouse gases/

7. carbon footprint/

8. environmental impact/

9. life cycle assessment/

10. water pollution/

11. waste disposal/

12. (climate adj2 change$).tw.

13. (planetary adj2 health).tw.

14. (environment$ adj2 (sustainab$ or emission$ or impact$ or cost$ or burden$ or wast$ or resource$ or conserve$)).tw.

15. (environment$ adj4 (manufactur$ or product$ or equipment or scrub$)).tw.
16. (unused adj3 (product$ or instruments or equipment or material$)).tw.
17. ((steriliza$ or reusable) adj5 (instruments or equipment$ or material$)).tw.
18. ("single use" adj5 (instruments or equipment or material$)).tw.
19. (waste adj3 (generat$ or audit)).tw.
20. (water adj2 (reduc$ or wast$ or modif$)).tw.
21. (carbon adj4 (emission$ or footprint$)).tw.
22. emissions.tw.
23. (greenhouse adj3 (gas$ or emission$ or effect$)).tw.
24. (global adj2 resource).tw.
25. or/4-24
26. 3 and 25
27. (animal$ or rabbit$ or rat or rats or mouse or mice or chicken$ or dog or dogs or cat or cats or feline or pig$ or monke$ or fish).ab.
28. 26 not 27
29. case reports/
30. (case adj2 (report$ or series)).tw.
31. 29 or 30
32. 28 not 31
33. (spectral or photon or otoacoustic or fluorescence).tw.
34. (cell$ or mutation$ or genes or genome or sequencing).tw.
35. 33 or 34
36. 32 not 35
